# Oligodendrocyte Precursor Cells in Spinal Cord Injury: A Review and Update

**DOI:** 10.1155/2015/235195

**Published:** 2015-09-27

**Authors:** Ning Li, Gilberto K. K. Leung

**Affiliations:** Department of Surgery, Li Ka Shing Faculty of Medicine, The University of Hong Kong, Queen Mary Hospital, Pokfulam, Hong Kong

## Abstract

Spinal cord injury (SCI) is a devastating condition to individuals, families, and society. Oligodendrocyte loss and demyelination contribute as major pathological processes of secondary damages after injury. Oligodendrocyte precursor cells (OPCs), a subpopulation that accounts for 5 to 8% of cells within the central nervous system, are potential sources of oligodendrocyte replacement after SCI. OPCs react rapidly to injuries, proliferate at a high rate, and can differentiate into myelinating oligodendrocytes. However, posttraumatic endogenous remyelination is rarely complete, and a better understanding of OPCs' characteristics and their manipulations is critical to the development of novel therapies. In this review, we summarize known characteristics of OPCs and relevant regulative factors in both health and demyelinating disorders including SCI. More importantly, we highlight current evidence on post-SCI OPCs transplantation as a potential treatment option as well as the impediments against regeneration. Our aim is to shed lights on important knowledge gaps and to provoke thoughts for further researches and the development of therapeutic strategies.

## 1. Introduction

**Figure 1:**
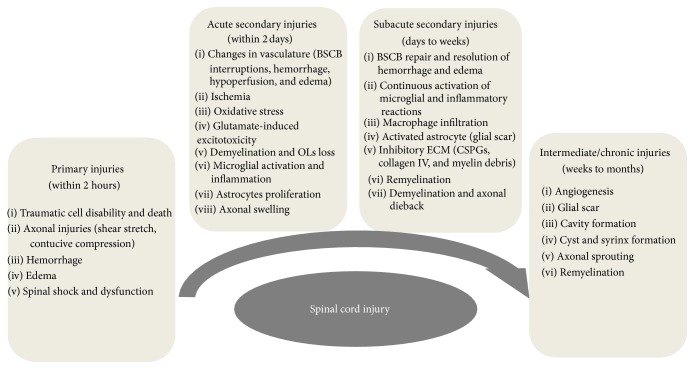
The major pathophysiological phases after spinal cord injuries. BSCB: blood-spinal cord barrier; OLs: oligodendrocytes; ECM: extracelluar matrix; CSPGs: chondroitin sulfate proteoglycans.

Spinal cord injury (SCI) is a catastrophic event that commonly results in axonal injuries and deaths of neurons and glial cells. Subsequent secondary injuries that consist of uncontrolled inflammation, excitotoxicity, edema, ischemia, and chronic demyelination can lead to additional damages, while the formation of glial scars also prohibits axonal regeneration [1] (Figure 1). SCI causes disturbances to normal sensory, motor, or autonomic functions and can significantly affect patients' physical, mental, and social well-being [2, 3]. Current therapies mainly rely on early operations for mechanical decompression, symptomatic relief, supportive care, and rehabilitation. With the development of stem cell technologies, cell-based transplantation is now thought to be a promising therapeutic approach for SCI. In fact, an autologous bone marrow stem cell transplantation approach is already undergoing a phase II clinical trial (NCT02009124, https://clinicaltrials.gov/), while a neural stem cell transplantation study is currently in phase I/II trial (NCT02326662, https://clinicaltrials.gov/). Though exciting, their clinical utilities are still far from being clear partially due to unclear safety issues such as teratoma formation.

A potentially useful cell source for post-SCI transplantation is oligodendrocyte precursor cells (OPCs). The latter are the major source of oligodendrocytes responsible for myelination within the central nervous system (CNS). The proliferation, migration, and differentiation of OPCs are sophisticatedly regulated by numerous factors including neuronal- or axonal-glial neurotransmitters, growth factors, neurotrophins, and transcription factors. The majority of OPCs are quiescent with limited self-division under normal circumstances, but they may respond rapidly to injuries and, in particular, demyelination. However, their rescuing effects are commonly hindered by the hostile microenvironment at the injury sites, leading to incomplete remyelination and clinical recovery. Therefore, finding ways to boost endogenous OPCs by enhancing the positive regulatory factors while attenuating negative ones has been an area of intense investigations in neurotrauma research.

This review will first summarize known characteristics of OPCs and then focus on the current understandings about the potential roles of OPCs in SCI, in particular, their effects on remyelination and glial scars formation. Recent progress in OPCs transplantation research and associated concerns will be discussed as well. Our aim is to shed lights on important knowledge gaps and to provoke thoughts for further researches and therapeutic treatment strategies.

## 2. Oligodendrocytes Loss and Demyelination after Spinal Cord Injury

The myelin sheaths are essential for saltatory signal conduction and tropic support to maintain axonal integrity [4]. Unfortunately, mature oligodendrocytes, the only myelin-forming cells within the CNS, are highly susceptible to damages [5]. Grossman et al. observed an acute loss of oligodendrocytes, along with neuronal death, as early as 15 minutes after injury in a rat spinal contusion model [6] and which might last for 3 to 7 days [7]. In an observational study with a 450-day follow-up after contusive SCI in adult rats, the extent of demyelination significantly dropped within one week after injury, followed by fluctuations at a lower level for about 70 days, and then increased steeply during the rest of the observation period. The findings suggested a chronic on-going process of aggravated demyelination [8].

The underlying mechanisms are far from clear, however. Besides the initial acute insults, both necrosis and apoptosis of oligodendrocytes have been observed in the chronic phase of injury [9–12]. Numerous factors may contribute to this process including the overabundant release of proinflammatory cytokines such as tumor necrosis factor-*α* (TNF-*α*) and interleukin-1*β* (IL-1*β*), uncontrolled oxidative stress, and ischemia as well as glutamate- and ATP-mediated excitotoxicity [12, 13]. It is worth pointing out that though autophagy, another type of cell death, has been shown to cause oligodendrocytes death [14, 15], its positive and negative impacts remain controversial. Smith et al. demonstrated that autophagy did not increase oligodendrocyte loss in the spinal cord upon terminal deoxynucleotidyl transferase dUTP nick end labeling (TUNEL) assay and caspase-3 immunostaining; instead, autophagy was found to promote myelin development [16].

Historically, it was thought that oligodendrocytes and intact myelin sheath were primarily responsible for the facilitation of neuronal signal conduction only. The potential role of oligodendrocytes in preserving the integrity and survival of axons was not realized until Griffiths and McCulloch reported acute retraction of some lateral loops from paranode at 1.5 hours after injury due to oligodendrocytes loss [17]. Given the fact that each oligodendrocyte is responsible for 30–80 distinct axons, it could be expected that extensive demyelination may occur even after the collapse of only a single oligodendrocyte [18, 19]. Indeed, transgenic mice with mutated myelin proteins such as myelin proteolipid protein (PLP) and DM-20 were found to show overt axonal swelling and degeneration [20]. Similar observations were also made in mice harboring mutations of other mature-oligodendrocyte-related proteins or genes [21–23]. Together, these findings have given rise to the current concept that axonal integrity relies heavily on oligodendrocyte support and that oligodendrocyte loss would result in axonal degeneration.

It is known that axons of cortical neuron may project as far as 100 cm within the corticospinal tract [24]. Besides, myelin sheaths essentially shield axons from their surroundings and limit access to extracellular metabolites. These raise concerns about metabolic homoeostasis and energy supply to the axons. In this regard, oligodendrocytes have been shown to produce lactate, an alternative energy source for axons within the CNS [25–27]. Reducing MCT-1, one of the monocarboxylate transporters (MCTs) in oligodendrocytes, resulted in severe axonal swelling, indicative of the important role of oligodendrocyte-derived lactate [28]. Furthermore, myelinating oligodendrocytes were able to synthesize and deliver ATP to axons through connexons, a kind of gap junctions protein [29, 30]. This increases the conduction speed of action potentials [31]. Other factors responsible for axonal development and stabilization include 2′,3′-cyclic nucleotide phosphodiesterase 1 (CNP-1) gene [32] and peroxisomal targeting signal 1 receptor (PEX5) gene [33]. These findings suggest that, in addition to their predominant functions in nerve conduction, oligodendrocytes may also labor as mechanical and trophic supports to axons [24, 26]. As naked or demyelinated axons are more vulnerable to injuries, it is reasonable to expect that, after injury, efficient remyelination is critical not only for cellular replacement but also neuron-glial cross talk reconstruction and neuronal function recovery.

## 3. Oligodendrocyte Precursor Cells and Their Characteristics

### 3.1. Multipotency of Oligodendrocyte Precursor Cells

Vaughn and Peters first discovered a type of small and irregularly shaped cells with stout processes that were without astrocytes or oligodendrocytes characteristics in adult, embryonic, and perinatal rat optic nerves [49]. On autoradiography, these cells reacted to Wallerian degeneration and represented 85–90% of proliferating cells after degeneration despite the fact that they only accounted for ~5% of all glia under normal conditions [50, 51]. These cells were initially termed “oligodendrocyte-type-2 astrocyte (O-2A) progenitor cells” due to their potencies for developing into either oligodendrocytes or type-2 astrocytes under specific conditions [52–54]. More recently, these cells were renamed “oligodendrocyte precursor cells (OPCs)” because of their predominant function of regenerating oligodendrocytes in demyelinating conditions [55]. OPCs are found in both white and gray matters within the CNS though abundantly so in the former [56]. Morphologically, OPCs have small cell bodies and multiple processes and may adopt a bipolar or tripolar shape [57].

Interestingly, the terminology of OPCs has received further challenges because of recent discoveries of their abilities to give rise to neurons. Purified OPCs from postnatal day 6 rat optic nerve have been shown to revert back to multiple CNS stem cells, which could in turn give rise to neurons and oligodendrocytes as well type-1 and type-2 astrocytes [58]. Although these findings were initially challenged on the ground of experimental techniques [59], they were subsequently confirmed both* in vitro* and* in vivo* [60, 61]. Guo et al. detected low expression of doublecortin (DCX), a marker for migrating and immature neurons, in a population of cells with negative HuC/D signals (exclusively presented in neurons) but positive PDGFR-*α* and Sox10 signals, both of which are determinating markers of final oligodendrocyte maturation [62]. These DCX+/PDGFR-*α*+/HuCD- cells raised thoughts about the idea that at least some endogenous OPCs do have characteristics of neurons. Indeed, using PDGFR-*α* promoter-driven Cre, scientists were able to induce neuronal formation from OPCs in adult piriform cortex [63]. Similar findings were also observed* in vivo* within adult rat neocortex [64], neonatal mouse forebrain [65], and postnatal cerebral cortex [62].

### 3.2. OPCs as Postsynaptic Neuronal Regulatory Targets

The conventional dogma that classic chemical synapses exist exclusively as neuron-neuron connections was challenged by the discovery of functional glutamatergic synapses between OPCs and axons [66–69]. The latter would include N-methyl-D-aspartate receptor (NMDAR), alpha-amino-3-hydroxy-5-methyl-4-isoxazolepropionic acid receptor (AMPAR), and kainite receptors. On oligodendrocytes, these receptors are activated by glutamate released by either neurons or axons, which increases intracellular Ca^2+^ level through stimulation of voltage-dependent Ca^2+^ channels or the reversal of Na^+^/Ca^2+^-exchangers. Li et al. demonstrated that NMDA would promote OPCs maturation and myelination in dorsal root ganglions- (DRGs-) OPCs coculture system* in vitro *[69]. Moreover, glutamate released by cultured cortical neurons could promote OPCs migration by stimulating NMDAR; T lymphoma invasion and metastasis 1 (Tiam1), a Rac1 guanine nucleotide exchange factor (Rac1-GEF) that is coexpressed and interacts with NMDAR in OPCs, would antagonize NMDAR and suppress migration [70].

Remyelination was found to be significantly delayed by the NMDAR specific antagonist MK801 in an* in vivo* cuprizone demyelination model, suggesting that NMDAR is essential for the entire OPCs-initiated remyelination process [69]. Others such as AMPA-type glutamate receptors may also participate in neuron- and axon-OPCs connections. AMPAR activation appeared to be essential for OPCs morphological alterations rather than proliferation and differentiation during myelination [71]. AMPAR blockade resulted in the inhibition of OPCs morphological development while promoting proliferation and differentiation [72]. Interestingly, a recent study also demonstrated that the degree of OPCs-neuron contacts actually differed between demyelination and remyelination phase in that synapses would only form during remyelination upon sufficient proliferation after demyelination [73]. These findings were derived from a lysolecithin- (LPC-) induced demyelination model, however, and whether the same would apply in SCI remains to be tested. It is plausible that OPCs may lack glutamatergic synapses immediately after injury in order to promote self-proliferation, and synaptic inputs would only develop during the migration and maturation stages in preparation for further remyelination. And to accomplish final myelin sheaths formation, a subsequent regain of AMPA synapses may be required for correct morphological development.

More than 90% of synaptic currents in OPCs in the neocortex are evoked by gamma-aminobutyric acid-ergic (GABAergic) synapse [74], and GABAergic connections between neuronal axon and OPCs are abundant [75, 76]. As in the case of glutamatergic receptors, activation of GABA-*α* receptors may trigger Ca^2+^ influx via Na^+^/Ca^2+^ exchangers and promote OPCs migration [77]. In light of this evidence, it is highly likely that Ca^2+^ influx triggered by either glutamatergic or GABAergic synapses is essential for OPCs migration by means of cytoskeletal reorganization, cell mobility, membrane traffic, and cell adhesion and deadhesion [78]. On the other hand, a more complex case is found in a cerebellar diffuse white matter injury model in which hypoxia-induced loss of GABA-*α* receptor-mediated synaptic input to OPCs significantly suppressed OPCs maturation while promoting proliferation [79]. In this regard, it seems that depolarization or hyperpolarization of GABAergic synapses contributes to different aspects of OPCs regulation. The expression of GABA-*β* receptors on OPCs and their physiological functions is incompletely understood. It is known that both GABA-*β*1 and GABA-*β*2 subunits are localized on OPCs and that stimulating GABA-*β* receptors by baclofen, a selective agonist, substantially increases migration and proliferation, providing evidence for a functional role of GABA-*β* receptors in oligodendrocyte development [80]. The temporal-spacial alteration of either type of GABAergic synapses after CNS injury and the effect on OPCs and posttraumatic remyelination are not yet clear.

Purinergic receptors, including P1 receptors (alternatively termed as adenosine receptors), metabotropic P2Y receptors, and ionotropic P2X receptors, are widely distributed in neurons and glial cells. As the breakdown product of ATP, adenosine is the main ligand targeting four subtypes of adenosine receptors (A1, A2a, A2b, and A3 receptors), all of which have been identified in OPCs [81]. Activation of adenosine receptors evokes the alteration of Ca^2+^ signals via G-protein coupled receptors in OPCs and regulates their differentiation and myelination. Particularly, activation of A1 receptor may be indispensable in recruiting OPCs to sites of injury where remyelination occurs [82]. Contrarily, A2 receptors may possibly act in a different way since they simulate cyclic adenosine monophosphate (cAMP) via G_s_ receptors rather than G_i_ receptors as for A1 receptors. Indeed, selective A2a receptor activation may suppress OPCs proliferation* in vitro* through the inhibition of K^+^ currents [83]. Though little knowledge is provided yet, it is highly believed that figuring out the effects of A2b receptors upon OPCs may offer more information in regulating them under pathological circumstances since it is shown that higher concentration of adenosine is needed for activation of A2b receptors, which probably can be detected in trauma, hypoxia, or ischemia [84].

Adenine (i.e., ATP and ADP) and uracil nucleotides (i.e., UTP and UDP) activate P2 receptors rather than adenosine receptors. Activating P2X7, the only functionally active P2X receptor in OPCS, results in a sustained influx of Ca^2+^ and regulates pathological responses of OPCs [85]. There is strong evidence that blocking P2X7 can have a neuroprotective effect against ATP-induced oligodendrocytes-toxicity [86] and that downregulation of P2X7 receptors expression may occur after hypoxic ischemic injury [87]. While metabotropic P2Y receptors may or may not be directly related to cell viability, their involvements in promoting OPCs migration [88] and prohibiting proliferation [85] have been reported. The functions of other P2Y receptors subtypes such as P2Y2, P2Y4, and P2Y12 in OCPs and SCI are even less well understood and deserve further investigations [89, 90].

The expression of muscarinic acetylcholine (mACh) receptors, a subtype of cholinergic receptors, has been successfully identified on OPCs [91]. Activation of mACh may rescue OPCs from growth factor deprivation [92]. Alternatively, mACh receptors activation significantly promotes OPCs proliferation while prohibiting their maturation [93]. Conversely, antimuscarinic adjunct therapy was found to offer a prodifferentiative effect in human OPCs* in vitro*. Enhanced functional recoveries were also shown with systemic treatment with mACh receptors antagonist following transplantation of human OPCs into hypomyelinated rats [94]. The role of nicotinic acetylcholine (nACh) receptors, which are expressed on OPCs also, is not yet known [95, 96].

### 3.3. Regulation of Oligodendrocyte Precursor Cells

OPCs functions are intricately modulated by a complex network of growth factors, cytokines and chemokines, neurotrophins, and transcription factors, all of which would affect the remyelinating process in SCI.

*Growth Factors.* Platelet-derived growth factor-A (PDGF-A) and fibroblast growth factor-2 (FGF-2) are two classic factors affecting oligodendrocyte lineage development. Stable expression of PDGFR-*α* in OPCs and the mitogenic effect of PDGF-A, a ligand to PDGFR-*α*, in oligodendrocyte lineage have been long established [57]. OPCs in white matter respond to PDGF-A stimulation via Wnt/*β*-catenin and phosphatidylinositol 3-kinase pathways by increasing proliferation rate [97]. The evidence that PDGF-A stimulus may accelerate the migration of OPCs by activating ERK pathway gives thought to its potential prorecruitment role on OPCs after SCI [98]. FGF-2 is another strong mitogen to OPCs. Direct intraventricular injection of FGF-2 after subventricular zone dissection injury not only enhanced the generation of OPCs but also promoted the differentiation of oligodendrocytes from OPCs [99]. In agreement with this, a double knockout of FGF receptors 1 and 2 significantly impacted on the differentiation of OPCs and hindered myelin sheaths formation in a chronic cuprizone-induced demyelination model but not in acute demyelination in the same study [100]. Interestingly, proliferation of OPCs can be induced in culture of conditioned medium from B104 neuroblastoma cells, possibly due to the coexistence and synergy of PDGF-A and FGF-2 [101]. Other growth factors include insulin-like growth factor (IGF), which has synergic functions with PDGF and FGF through common downstream messengers like PI3K/AKT and ERK pathways.

Ciliary neurotrophic factor (CNTF), a pleiotropic cytokine within the IL-6 family, has been shown to promote spinal cord-derived OPCs survival and differentiation in culture as well as endogenous OPCs migration in an acute demyelination model [102, 103]. In the situation of SCI, a chronic and continua increase of CNTF level was observed in contusive spinal cord tissues, especially along the border of injury site where posttraumatic oligodendrogenesis was severe [104]. The idea of CNTF being involved in the regulation of OPCs after SCI is supported by a recent study showing enhanced remyelination and functional recovery after transplantation of CNTF-expressing adult OPCs into contused spinal cord [34].

*Neurotrophins.* Neurotrophins are responsible for the regulation of survival and development of neural cells including neurons and progenitor cells. Direct promyelinating effect of brain-derived neurotrophic factor (BDNF) via activation of TrkB receptors has been demonstrated in dorsal root ganglions- (DRGs-) OPCs coculture system [105]. Consistently, deletion of TrkB receptors in mice reduced thickness of myelin sheath but would have no effect on oligodendrocyte maturation or the number of myelinated axons. Surprisingly, knockout of TrkB receptors actually led to a significant increase in the density of OPCs, suggesting that the regulatory effects of BDNF via TrkB receptors may be phase-dependent [106]. Neurotrophin-3 (NT-3) is another neurotrophin that has a positive role in inducing survival and proliferation of OPCs [107]. Both BDNF and NT-3 expressions increase after SCI in mammals [108, 109], and additional NT-3, alone or combined with sonic hedgehog, could increase the number of OPCs and enhance remyelination after SCI [110].

*Chemokines and Cytokines.* Chemokines are key players in the immunological and hematological systems. They are also coexpressed with neurotransmitters such as choline and dopamine in specific regions of the brain where they serve specific functions [111, 112]. Though the expression of a number of chemokines would alter after SCI, not all are responsible for OPCs modulation. For example, CCL2 (also known as MCP-1) expression is elevated during the acute phase after spinal cord damaging and its inhibition would attenuate secondary SCI [113]; it does not affect proliferation and myelination of OPCs [114]. In contrast, CXCL12 (SDF-1*α*) and its receptor CXCR4 would promote OPCs proliferation and myelin sheath formation in a dose-dependent matter [114–116]. CXCL1 (GPO-*α*) and its receptor CXCR2 may also take part in the regulation of OPCs survival in pathological states. Enrichment of CXCL1 ameliorates OPCs death from inflammation cascade, which is a major component of secondary injury after CNS trauma [117, 118].

It has been demonstrated that the synthesis of several cytokines such as leukemia inhibitory factor (LIF) is upregulated after SCI [119]. LIF is known to promote oligodendrocyte lineage development [120] and protect OPCs after demyelinating injuries and ischemic trauma [121, 122]. Also, transportation of LIF through impaired spinal cord-blood barrier has been observed after SCI [123]. Interferon-gamma (IFN-*γ*) also exerts interesting dual effects on OPCs in a concentration-dependent manner; it maintains OPCs in the cell cycle at low level of expression, whilst higher levels would cause demyelination [124]. IL-17A is another new candidate that can induce OPCs to exit the cell cycle and commence differentiation into mature oligodendrocytes by activating ERK1/2 pathways in demyelinating pathologies [125]. These effects may occur in conjunction with IL-1*β*, which is known to protect OPCs and promote their differentiation. But unlike IL-17A, IL-1*β* arrests OPCs in the cell cycle and hinders their mitosis [126]. It would be interesting to study the combined effects of IL-17A and IL-1*β* upregulation after SCI [119].

*Transcription Factors.* A vast body of literatures has described the transcriptional networks that regulate OPCs, one of which is the basic helix-loop-helix (bHLH) family. Of particular interest is the significant role played by oligodendrocyte transcription factors 1 (OLIG1) and OLIG2, both being oligodendrocyte-specific genes [127, 128]. Double-mutant* OLIG1* and* OLIG2* would eliminate the formation of OPCs and the genesis of motor-neurons and oligodendrocytes [129], while transient expression of* OLIG1* would induce the formation of OPCs from neural stem cells [130]. For remyelination, a series of loss- and gain-of function* in vitro* studies have demonstrated the comparatively more predominant role of* OLIG2* [131–133]. Overexpression of* OLIG2* in transgenic mice may lead to precocious myelination throughout the CNS as well as enhanced OPCs migration and remyelination [134].* OLIG1* and* OLIG2* also interact. While* OLIG2* expression is upregulated in contused spinal cord [135], its solo overexpressionwould result in tumorous cell growth. Interestingly, such tumorigenesis is absent under the situation of simultaneous expressions of* OLIG1* and* OLIG2*, indicating a directly restrictive and mutually modulatory function between them [136]. Compared with* OLIG2*,* OLIG1* plays a secondary and nonessential role in oligodendrogenesis as* OLIG1* null mice lines have shown only a mild delay in oligodendrocyte differentiation and maturation without long-term effect [137].

OLIGs are modulated by other transcription factors such as inhibitor of DNA-binding protein 4 (ID4) and its companion ID2, both of which act as overt transcriptional repressors upon OPCs differentiation and maturation by sequestrating OLIG1 and OLIG2 and preventing them from binding consensus DNA domains [138, 139]. This indicates a potential modulating role of IDs on OPCs in SCI. Indeed, electroacupuncture treatment after compressive spinal cord injury was found to protect myelin sheath breakdown and increase OPCs proliferation by promoting OLIG2 but attenuating* ID2* expression [140].

The high mobility group (HMG) family, another group of transcriptional factors, also modulates OPCs. In particular, SOX5 and SOX6 from D subgroup of HMG are found in OPCs, and they repress the terminal maturation of OPCs and hinder their migration [141]. In OPCs,* SOX6* expression requires the consistent presence of SOX8 and SOX9, which guarantee the generation of OPCs in conjunction with SOX10 [141, 142]. The latter is crucial for the terminal differentiation of OPCs since ablation of* SOX10* would lead to an altered migration pattern and reduced quantity of OPCs due to enhanced apoptosis [142]. SOX10 may also act as a regulating target and mediator of OLIG1 and OLIG2 during OPCs maturation [136]. The likely synergistic relationship between SOX10, OLIG, and MYRF further complicates the picture and calls for more detailed posttranscriptional studies [143, 144].

Current evidence also suggests the positive participation of ZFP191 and ZFP488, two members of zinc finger transcription factors, in OPC regulations. Mutation of* ZFP191* in C3H/HeJ genetic mice would lead to significant hypomyelination with preserved process-outreaching progenitors, indicating that ZFP191 may have a stage-specific action. ZFP488 is an oligodendrocyte-specific regulator. Its overexpression would promote oligodendrocyte precursor formation in cooperation with OLIG2 [145]. Of particular interest is myelin transcription factor 1 (MYT1) which is upregulated in the injured spinal cord [146]. In light of the evidence that MYT1 is crucial for progenitor proliferation and differentiation [147], it is possible that MYT1 may contribute to the modulation of OPCs in SCI. Many other transcription factors such as MASH1 (alternatively known as ASCL1) [148], NKX family [149], and Yin Yang 1 (YY1) [150] may similarly regulate oligodendrocytes generation although concrete evidence is still lacking.

## 4. Roles of Oligodendrocyte Precursor Cells after Spinal Cord Injuries

### 4.1. Alterations after Spinal Cord Injuries

OPCs respond to SCI rapidly by altering their morphologies and accelerating mitosis [151, 152]. Overall, the proliferation rate of OPCs would significantly increase by the end of the first day after injury and remain elevated in the following week. Cells number would remain high for a whole month. Two different subtypes of OPCs may be identified within and around the injury site [153]. Round-shaped OPCs with high NG2 staining and short thick processes were found along the injury lesions; this contrasts with OPCs with multiple processes seen 200–300 um away from the lesion. In general, OPCs positively stained for NG2 and BrdU antibodies were found throughout the impact site and the surrounding spared areas in the first week after injury [154]. Elevated proliferation continued for two weeks, followed by a decline within the epicenter and rostral sections but not the caudal parts. Another time-course study described a more rapidly upregulated proliferation by day 2 that peaked by the 4th day and then declined by week 1 [155]. A closer look at the temporal-spatial pattern showed that, instead of the epicenter, most BrdU+ OPCs were in fact concentrated within 1.5 mm rostral to the injury site 3 days after SCI [7, 156]; the number of cells labeled with NG2/Ki67 would drop within 4 hours, indicating a local toxic effect after injury [157].

There is hitherto scanty information on the mobility of OPCs after SCI although they are generally thought to migrate at a slower rate than their precursor stem cells [158]. Whether proliferative OPCs around the injury site are activated* in situ* or derived from immigrating cells remains unknown. Carroll et al. studied experimental demyelinating optic nerve lesions and reported a centripetal migration of cells towards the lesion by day four. Though not firmly identified as OPCs, these cells did differentiate into oligodendrocytes during and after migration [159], and it is possible that the same may occur in SCI. In another study using a spinal contusion injury model, treatment with the enzyme chondroitinase ABC, which removed the inhibitory effects of chondroitin sulfate proteoglycans (CSPGs), could significantly enhance the quantity of OPCs 2-fold [160]. Using double-staining techniques, the authors further demonstrated that none of the OPCs were positive for BrdU/Olig1 or Ki67/Olig1, suggesting that the increase of OPCs number was primarily due to migration rather than* in situ* proliferation.

### 4.2. Remyelination by Endogenous Oligodendrocytes Precursor Cells

Identifying the exact cell type responsible for remyelination in diseases is critical for the development of therapeutic interventions. Self-mitosis and replacement for the damaged cells are considered as a direct and plausible way of healing and recovery after injuries. As a consequence, mature oligodendrocytes, being the myelin-forming cells in the CNS, were initially thought to be responsible for posttraumatic remyelination [161]. However, this postulation was challenged by the evidence that terminally differentiated oligodendrocytes would represent no more than one-fourth of the total BrdU+ cells in spinal cord tissue following contusive injury [156], and surviving oligodendrocytes after injuries would possess extremely limited abilities to divide [162].

An alternative hypothesis is that OPCs may be responsible for the generation of myelinating cells and remyelination instead [152, 154, 163]. Watanabe et al. found a robust proliferation of OPCs during the first week after chemical-induced demyelination, followed by a steep decline of OPCs with an increase in the number of mature oligodendrocytes [164]. More importantly, a few thin myelin rings were detectable on day 14, and, by day 28, numerous myelin basic protein (MBP) positive myelin sheaths were observed throughout the lesion. The results indicated that new matured oligodendrocytes were mainly derived from OPCs and were able to produce myelin sheaths* in vivo*. Further supporting evidence is made available from transplantation experiments (see below). Cao et al. studied whether treatment with exogenous OPCs could alleviate demyelination and improve motor function [34]. Using OPCs expressing CNTF, grafted cells were found to differentiate into mature oligodendrocytes. Notably, transcranial magnetic motor-evoked potentials (tcMMEPs) and magnetic interenlargement reflex (MIER) showed a progressive recovery in both the CNTF-expressing OPCs group and the control OPCs group; no restoration was detected in nontransplantation groups. In another spinal cord irradiation injury model treated with mouse embryonic stem cells- (mESCs-) derived OPCs, a reduction in lesion volume and an increase in dorsal funiculus density were observed [39]. Forelimb locomotion improved in the transplantation group when compared with controls. These findings suggested that OPCs were able to generate mature oligodendrocytes, which may then become integrated and functional within the injury site.

It is important to note that OPCs may not be the only endogenous progenitors of myelinating oligodendrocytes after spinal cord trauma. Ependymal cells, located in the central canal of spinal cord, possess neural stem/progenitor cell properties and may respond extensively to insulting signals in spinal cord tissue. Such proliferative responses seem to be exclusive to spinal cord trauma and do not occur after chemical-induced demyelination [165]. Though, under normal circumstances, the proliferative activity of ependymal cells is only a tenth of that of OPCs, as soon as injury takes place, ependymal cells would produce as much as double the number of progenies compared to OPCs. Excitingly, a fraction of these cells would finally differentiate into functioning oligodendrocytes [166]. A recent study has demonstrated the cholinergic enhancement of cell proliferation on ependymal cells and, in turn, the upregulation in oligodendrocyte markers in spinal cord tissue [167], thus shedding lights on the possibility of endogenous oligodendrogenesis from ependymal cells.

Since the promising findings in the origins and sources of oligoregeneration and the wealthy understandings of endogenous regulation of OPCs have been stated, is the remyelination complete? Such a speculation is cruelly diminished by postmortem examinations providing disappointing results with the presence of substantial amount of demyelinated axons especially within the center of contused spinal cord tissues [168]. And, given the difficulties with distinguishing the boarder of injury epicenter and the chronic die back of naked axons, pathological examinations focusing on the injury site alone may potentially underestimate the actual number of dysmyelinated or demyelinated axons after spinal cord trauma. Spontaneous remyelination after CNS injuries therefore remains a highly elusive phenomenon. Recruitment of OPCs into injury site relies greatly on the involvement of multiple stimulating factors, the levels of which would diminish with age [169]. Another plausible reason for insufficient remyelination after injury may be the existence of myelin sheath debris containing inhibitory molecules against OPCs differentiation such as NOGO66 via NOGO receptor complex [170]. Myelin sheaths that have survived the initial insult may also inhibit the maturation of OPCs probably by upregulating the expression of* ID2* and* ID4* [171]. Furthermore, glial scars formation would hinder not only the recruitment and proliferation of OPCs but also create a hostile environment for OPCs differentiation and oligodendrocytes maturation. Paradoxically, OPCs themselves may also partake in scar formation, suggesting a dual role of OPCs after SCI (see below). Taken together, it is likely that myelin sheaths repair and oligodendrocyte lineage regeneration are hindered most significantly within the epicenter of injury, whilst a more optimal site for regeneration may be found along or even at a distance from the border of the injury [13].

Bone morphogenetic proteins (BMPs) belong to the transforming growth factor-*β* (TGF-*β*) super family. They act on type I and type II BMPs receptors and are important negative regulators in oligodendrocyte lineage generation [172]. Earlier evidence demonstrated that the levels of BMPs would alter after SCI [173]. Blockade of BMP4 by its extracellular antagonist, Noggin, prohibited astrogenesis and promoted the production of oligodendrocytes in OPCs culture [174]. Similarly, BMP4 was able to induce astrocyte generation and suppress oligodendrocytes production via the Smad pathways and other transcription factors [138, 173]. On the other hand, intraventricular infusion of BMP4 has been found to increase the number of OPCs during the demyelination phase. The number of OPCs significantly decreased with an increase in astrocytosis during remyelination 1 week later. Antagonism of BMP4 by Noggin could enhance remyelination and ablate the proastrogenesis effect of BMP4, suggesting that BMPs may act in a phase-specific fashion [174]. Understanding the role of BMP4 at different phases of repair is therefore of translational significance.

As mentioned above, undamaged myelin sheaths can inhibit remyelination via NOGO receptors. The latter interact with leucine-rich repeat and Ig domain-containing 1 (LINGO-1), which is a major suppressor of OPCs differentiation [175, 176]. Attenuating LINGO-1 significantly increased the survival of oligodendrocytes and improved functional recovery after spinal cord hemisection [177]. LINGO-1 may also function as a negative regulator of neurotrophin BDNF signaling through direct interaction with TrkB receptors [178]. Another negative regulator is tumor necrosis factors-alpha (TNF-*α*). Both TNF-*α* and its receptor, TNF-R1, are found in OPCs. Inhibition of TNF-*α* or TNF-R1 blockade could significantly attenuate the inhibitory effects of reactive astrocytes on OPCs differentiation at the epicenter of spinal cord [179].

Generally speaking, there are two major strategies that can potentially enhance remyelination: to increase the effects of stimulating factors and to block the effects of inhibitory factors. The manipulation of a single factor alone is, however, unlikely to the necessary effects, as exemplified by the failure to enhance remyelination in transgenic mice overexpressing PDGF-A despite an increase in OPCs density [180]. Some of the factors, such as BMP4 and ZFP191, may have dual effects that materialize in a phase-specific or region-specific fashion. Therefore, therapeutic strategies targeted at endogenous remyelination need to take into considerations the impacts of different treatment time points, site of application, and, most importantly, the combined effects of different factors. Currently, the use of exogenous cell source and biomaterial scaffolds have attracted considerable research effort.

### 4.3. Transplantation of Oligodendrocyte Precursor Cells

A growing body of evidence has demonstrated that cellular transplantation may be of benefits. Various kinds of transplantable cells, including olfactory ensheathing cells, Schwann cells, and stem cell-derived OPCs, have been studied [181]. The current understanding is that transplanted cells would offer not so much the replacement of damaged cells but the neuroprotective and immunomodulatory effects. Herein, we will highlight a few relevant studies.

#### 4.3.1. OPCs

Franklin et al. transplanted lac-Z-transfected O-2A progenitor cells (CG4, a cell line of OPCs) into irradiated spinal cord and observed cell survival, proliferation, and migration throughout the cord [35]. They further examined the reactions of CG4 cells in an ethidium-bromide induced demyelination lesion in both irradiated and nonirradiated spinal cords. By day 15 after injury, remyelination was evident microscopically in preirradiated demyelinated lesion. Interestingly, the injected cells appeared to survive better in an injured environment than in the normal spinal cord, indicating that damaged axons may emit factors that promote the survivals of OPCs. Exogenous OPCs transplanted directly into the injured site seven days after injury were found to have proliferated and differentiated into oligodendrocytes but not astrocytes or neurons. Both motor function tests and electrophysiological studies confirmed neurological recovery [36]. The therapeutic effects of transgenic OPCs were further demonstrated in SCI by Rosenbluth et al. [37] and Bambakidis and Miller [38]. A summary of these pioneering studies is provided in Table 1.

These preclinical studies have provided proof of concept evidence that exogenous OPCs could enhance remyelination, but whether they can provide clinical benefit remains unknown. The availability of autologous OPCs is limited and heterologous transplantation requires the use of long-term immunosuppression that may cause serious side effects [182]. A possible alternative is human embryonic stem cells (hESCs) which are capable of differentiating into oligodendrocytes.

#### 4.3.2. hESCs-Derived OPCs

hESCs are a potentially useful source of OPCs for clinical application [39]. They are also versatile, pluripotent “super cells” that are capable of differentiating into serotonergic [183] and dopaminergic neurons [184]. Nistor et al. described the method by which a population of OPCs with high purity and specificity could be generated from hESCs [185]. Using this technique, Keirstead et al. reported doubling of remyelinated axons one week after thoracic contusive SCI. Motor functions in the hESCs-OPCs-treated group were significantly better than those in controls [40]. The same research group also demonstrated that hESCs-OPCs transplantation was associated with better sparing of grey and white matter, attenuated cavitation, and altered gene expression (e.g., interleukin-10 (IL-10), hepatocyte growth factor (HGF), and Fas) in cervical spinal cord contusion [41]. In terms of somatosensory evoked potentials (SSEPs) as a surrogate of ascending pathway integrity [186], animals were found to have significant improvement in both amplitudes and latencies following transplantation [42].

These encouraging “proof-of-concept” studies have provided important evidence to support clinical trials. Recently, a company (Asterias) has obtained an approval from the US Food and Drug Administration to conduct a clinical phase I and phase II/a trial of OPCs transplantation (AST-OPC1) for SCI (NCT02302157, https://clinicaltrials.gov/). The trail is expected to commence in early 2015 and will test escalating dosages of transplanted cells and safety profile.

#### 4.3.3. iPSCs-Derived OPCs

OPCs may also be generated from induced pluripotent stem cells (iPSCs). iPSCs-derived OPCs provide the solutions for simultaneously resolving two major hurdles within transplantation therapy, that is, the scarcity of cell source and the problem of immune-rejection. iPSCs were derived from human dermal fibroblasts in 2007 [187]. Later, by genetically reprogramming several OPCs-related transcription factors (*OLIG2*,* SOX10*, and* NKX6.2*) in mouse fibroblasts cells, functional OPCs with normal morphology and oligodendrogenesis abilities were generated [188].

In 2011, Czepiel et al. succeeded in differentiating mouse iPSCs into OPCs and myelinating oligodendrocytes* in vitro* [44]. The majority of iPSCs-derived OPCs were found to have survived the procedure and could develop into functioning oligodendrocytes that participated in remyelination [45]. All et al. similarly transplanted iPSCs-derived OPCs into contused spinal cord and reported less cavitation together with enhanced axonal remyelination two months after treatment [46]. Perplexingly, both viable-OPCs group and Heat-killed-control group (one of the controls) showed functional improvement during the first seven days after transplantation. However, further improvement in the viable-OPCs group was not evident until the end of the first month. Notwithstanding, these studies have shown that iPSCs-derived OPCs are a potentially promising and clinically accessible cell source.

Douvaras et al. have reported an interesting study in which isolated skin fibroblasts from multiple sclerosis patients were induced to form iPSCs and later OPCs. These patient-iPSCs-derived OPCs were able to initiate myelination well after being transplanted into shiverer mice with myelin deficiency [47]. The limited migratory capacity of iPSC-derived OPCs derived from iPSCs could also be overcome by means of overexpressing polysialylating enzyme sialyltransferase X [48].

There are definite clinical risks associated with the use of stem cell-derived OPCs. The tumorigenicity is a major concern [189] even though no teratoma formation has so far been reported in Keirstead's serial studies [40, 41] or Geron's preclinical trial [190]. In another independent study, a total of 650 million hESCs-derived OPCs were injected, again, without any subsequent tumor-formation [43]. Another concern is that clinical and experimental SCI involve different pathological processes and that experimental models may fail to capture all critical elements found in clinical situations [191]. Whether derived OPCs are able to generate new oligodendrocytes and myelinate damaged axon is unknown. In addition, there are ethical considerations with regard to the use of human embryos that are beyond the scope of this review.

#### 4.3.4. Other Sources of Transplantable Cells

There are other types of transplantable cells that are potentially useful for the treatment of SCI including neural stem cells [192, 193], bone marrow cells [194, 195], and Schwann cells [196, 197]. The latter are the myelin-forming cells within the peripheral nervous system. The major advantages of Schwann cells include their accessibility and immune-compatibility. Their positive roles in myelinating regenerated axons, reducing cyst formation, and improving neural functions have been extensively studied. Another candidate for therapeutic transplantation is olfactory ensheathing cells. Preclinical studies have shown that these cells could promote remyelination [198, 199], and autologous olfactory ensheathing cells are currently being studied in clinical trials with some encouraging results [199–202]. In a recent study using autologous olfactory lamina propria transplantation, half of the patients with complete SCI showed some improvement in either motor or sensory functions upon long-term follow-up although the findings are not conclusive [203]. The authors tried to link several reasons to their results, one of which was the relatively small size of input graft when compared with the lesion size. Indecipherable enlargement of syringomyelia might also contribute to the mere recovery in this study. From our point of view, another plausible reason may be the long time span since spinal cord trauma (not less than 6 months), as it is indicated that benefits may not be witnessed when treatment is carried out after a several months-long period of time [40].

### 4.4. Inhibition by Oligodendrocyte Precursor Cells

Astrogliosis and glial scar formation are ubiquitous findings in SCI [204–206]. Although they serve important functions during the acute and subacute phases of injury, chronic scarring would limit axonal regeneration [207, 208]. The observation of extensive OPCs proliferation along injury boundaries also raises the question of whether these cells may in fact contribute to scarring. There are two main areas within a glial scar—the fibroblastic core and the glial surrounding zone [209]. NG2-positive cells were known to populate lesion cavities 2 days after injury, and, by day 10, these cells formed small plaques that corresponded to the glial surrounding zone. Similarly, oligodendrocytes precursors have also been identified within the fibroblastic core [210]. Hence, there is evidence to suggest that OPCs may also partake in the formation of glial scar that prohibits axonal regeneration.

Apart from being a physical barrier against regeneration, a glial scar may also act as a source of inhibitory molecules, including chondroitin sulfate proteoglycans (CSPGs), semaphorin 3, and eph/ephrin tyrosine kinases [211]. CSPGs consist of several subtypes (e.g., aggrecan, versican, NG2, neurocan, brevican, and phosphacan) and are closely linked with OPCs [212, 213]. Amongst these, NG2 is of particular interests. Chen et al. found that growing neurites would avoid OPCs-covered areas* in vitro*. But when incubated with anti-NG2 antisera, these OPCs-coated membranes would attract neurite ingrowth, suggesting that NG2 may be responsible for the growth-inhibiting effect of CSPGs [214]. The underlying mechanism is incompletely understood but may involve the activations of the N-terminal globular domain (domain 1) and the juxtamembrane domain (domain 3) of NG2 [215]. Results from subsequent* in vivo* studies were less clear-cut, however. de Castro et al. compared between wild-type and NG2 knockout mice and found no difference in the morphology of the transection scar. Surprisingly, calcitonin gene-related protein-positive fibers were found to grow into the scars in wild-type animals just as extensively as in NG2-null ones [216]. On the other hand, blockage of NG2 has been shown to facilitate the growth of ascending sensory axons across the caudal boundary of a lesion [217]. Similar growth-promoting effects could also be achieved by using chondroitinase ABC [218] and NG2 neutralizing antibodies [219]. There is as yet no satisfactory explanation for these controversial findings but differences in regenerating capabilities between subtypes of neurons may be responsible.

Recent studies also suggested that CSPGs might affect the formation of cellular processes by OPCs and oligodendrocytes. In an* in vitro* study, the presence of CSPGSs was found to reduce the length and number of processes in predifferentiated OPCs [220]. Furthermore, CSPGs could affect myelination, resulting in fewer membrane sheets that were smaller and atrophic [221]. Both Rho-Rock and protein kinase C signaling pathways [221], as well as the protein tyrosine phosphatase sigma (PTP*σ*) pathway [222], were thought to play important roles. Conversely, the reduction of CSPGs by xyloside was found to result in a robust increase in OPCs number as well as CC1+ mature oligodendrocytes in a mouse model of lysolecithin-injected spinal cord injury [223]. To further complicate the picture, different classes of CSPGs may exert different effects on OPCs [160]. Therefore, further researches are clearly needed.

## 5. Conclusions

Demyelination and oligodendrocyte loss following SCI cause significant interruption of neural functions. Current evidence supports the notion that efficient oligodendrocyte replacement and sufficient remyelination would ameliorate pathological cascades and improve neural functions. OPCs are the natural source of myelinating oligodendrocytes within the CNS following SCI. The enhancement of endogenous regeneration by innate OPCs and the transplantation of myelinating cells represent the two most promising therapeutic strategies. The elusive biological properties of OPCs and the complexity of the associated regulatory network are incompletely understood and continue to be a critical area of future investigations. A simplistic approach of targeting a single factor is unlikely to provide significant clinical benefit. The latter would necessitate a far more thorough understanding and exploitation of the complex regulatory network.

In the context of transplantation, OPCs derived from different sources may respond differently to individual growth factors and within different regions around the injury site [97, 101]. More intriguing is the possible role of OPCs in promoting glial scar formation that may itself inhibit axonal regeneration, and it is conceivable that future discoveries may argue against the utilization of OPCs transplantation in the treatment of SCI and other demyelinating conditions. The situation is further complicated by the fact that CSPGs may in turn prohibit OPCs. Existing evidence suggests that adult OPCs contribute to CSPGs accumulation at the lesion site after injury and that premature oligodendrocytes probably do not [213]. This renders premature oligodendrocytes an attractive source of transplantable cells that are capable of generating mature oligodendrocytes without emitting CSPGs. Again, given the extremely complicated neuron-glial, glial-glial, and extracellular matrix-glial cross talks, it is unlikely that single-agent treatment would be effective; instead, combinatorial strategies targeted at oligodendrocytes lineage protection, blockage of extracellular inhibitory molecules, and myelination promotion are probably necessary.

## Figures and Tables

**Table 1:** The summary of OPCs transplantation-associated literatures within the review.

References	Cells utilized	*In vivo* models	Transplantation strategies			Results
			Time point	Quantity	Location	
Cao et al. [34]	CNTF-expressing OPCs and regular OPCs	Contusive spinal cord injury (T9)	8 days after injury	4 × 10^5^	1 mm cranial and caudal to the lesion; left and right of the lesion	Implanted OPCs develop into mature oligodendrocytes tcMMEPs and MIER confirm a progressive recovery in both CNTF-expressing and regular OPCs, though it is more significant in the former group

Franklin et al. [35]	LacZ labeled CG4 cell line	X-irradiation pretreatment (T12 to L4) Ethidium bromide-induced demyelination	—	—	Lesion center; adjacent areas; remote areas	Cells survive, migrate, and are remyelinated better in irradiated cords than nonirradiated cords In nonirradiated cords, adjacently transplanted cells contribute to remyelination, whereas remotely transplanted cells do not

Lee et al. [36]	O-2A cells from P2 rat brain	Contusive spinal cord injury (T9)	7 days after injury	5 × 10^5^	Lesion center	Transplanted cells survive and differentiate into oligodendrocytes but not astrocytes or neurons A significant improvement in hindlimb performance There are no differences in SSEPs study, but the latency of MEPs is shorter in transplantation group

Rosenbluth et al. [37]	Transgenic mice with LacZ gene under control of MBP promoter	Contusive spinal cord injury (T9 to T10)	1 day–16 days after injury	1 × 10^6^	Lesion center	Cells move rostrocaudally over considerable distances and more readily to demyelinated areas Some of the cells succeed in myelin formation

Bambakidis and Miller [38]	OPCs from P0 rat spinal cords	Contusive spinal cord injury (T9 to T10)	5 days after injury	1.5 × 10^5^	Lesion center	Transplantation of OPCs with or without SHH improves axonal conduction (MEPs) and hindlimbs motor function The benefits seem more pronounced with the addition of SHH

Sun et al. [39]	mESCs-derived OPCs	Irradiation spinal cord injury (C4-C5)	4 months after irradiation	2 × 10^5^	4 mm cranial and caudal to the irradiated site	Transplanted mESCs-derived OPCs survive, migrate, and differentiate into oligodendrocytes within the irradiated lesion Histological examination shows a narrowed cavitation and a dorsal funiculus with increased densities Locomotion of fore limbs is improved in transplantation group

Keirstead et al. [40]	hESCs-derived OPCs	Contusion spinal cord injury (T10)	7 days and 10 months after injury	1.5 × 10^6^	4 mm cranial and caudal to the lesion center	7-day group: OPCs survive, differentiate into oligodendrocytes, and remyelinate axons; BBB scores are significantly higher in OPCs-treated rats 10-day group: OPCs survive and differentiate into oligodendrocytes but do not participate in remyelination; there is no improvement in BBB scores

Sharp et al. [41]	hESCs-derived OPCs	Contusion spinal cord injury (C5)	7 days after injury	1.5 × 10^6^	Cranial and caudal to the lesion center (interval is unknown)	Transplanted cells survive, redistribute, and differentiate in the injury sites; OPCs-remyelination efficiency is much higher BBB scores, forelimb stride length, and range of motion are improved significantly OPCs transplantation improves axon sparing and attenuates cavitation; it also alters the injury-induced gene expression (IL10, Fas, HGF, etc.)

All et al. [42]	hESCs-derived OPCs	Contusion spinal cord injury (T8)	2 hours after injury	1 × 10^6^	Lesion site; 4 mm cranial and 1 mm left; 4 mm caudal and 1 mm right	Transplanted cells survive and differentiate into myelinating oligodendrocytes while no astrogenesis is observed OPCs transplantation shows improvement in SSEPs amplitudes and latencies Cavitation in treated group is attenuated, and LFB staining is much higher

Kerr et al. [43]	hESCs-derived OPCs	Contusion spinal cord injury (T8)	3 and 24 hours after injury	1.5 × 10^5^ 5 × 10^5^, respectively	T7 and T9 And T8	Transplanted cells survive and migrate well without tumor or cyst formation Behavioral and electrophysiological examination improves in the OPCs-treated group

Czepiel et al. [44]	iPSCs-derived OPCs	*In vitro*: coculture of iPSCs-derived OPCs and DRGs *In vivo*: cuprizone-induced demyelination mouse model	—	1 × 10^5^	Corpus callosum	*In vitro*: extensive myelin formation around naked axons *In vivo*: 80% cells do not survive the injection, while survived ones develop into mature oligodendrocytes Teratoma is seen in rats treated with cells containing undifferentiated iPSCs

Pouya et al. [45]	iPSCs-derived OPCs	Optic chiasm demyelination by lysolecithin	1 week after lysolecithin administration	2 × 10^5^	Chiasm	A reduction in latencies of VEPs in transplantation group is seen Transplanted OPCs integrate and differentiate into oligodendrocytes

All et al. [46]	iPSCs-derived OPCs	Contusion spinal cord injury (T8)	24 hours after injury	5 × 10^5^	Lesion site	OPCs transplantation reduces cavitation, scars formation, and microglial proliferation Transplanted OPCs differentiate and are remyelinated in the lesion BBB scores improvement is only significantly seen after the first month

Douvaras et al. [47]	OPCs induced from iPSCs derived from MS patients (hiPSCs-derived OPCs); hESCs-derived OPCs	Shiverer/rag2 mice	—	1 × 10^5^ (5 × 10^4^ each side)	Bilaterally at a depth of 1.1 mm into the corpus callosum	OPCs can be efficiently generated from hiPSCs Very few hiPSCs-OPCs differentiate into astrocytes, and no neurons are found Transplanted hiPSCs-OPCs are myelinated in the brain

Czepeil et al. [48]	iPSCs-derived OPCs with overexpression of STX	*In vitro*: coculture of iPSCs-derived OPCs and DRGs *In vivo*: cuprizone-induced demyelination mouse model	—	1 × 10^5^	Corpus callosum	STX-treated OPCs show a significantly increased migratory ability *in vitro* and *in vivo* Survival and maturation pattern of STX-treated and control OPCs are similar

BBB scores: Basso, Beattie, and Bresnahan scores; CNTF: ciliary neurotrophic factor; DRGs: dorsal root ganglion neurons; HGF: hepatocyte growth factor; IL-10: interleukin-10; LFB: Luxol fast blue; MBP: myelin basic protein; MEPs: motor evoked potentials; SHH: sonic hedgehog; STX: sialyltransferase X; SSEPs: somatosensory evoke potentials; tcMMEPs: transcranial magnetic motor-evoked potential; VEPs: visual evoked potentials.
